# Novel Neuroprotective Lead Compound Ligustrazine Derivative Mass Spectrometry Fragmentation Rule and Metabolites in Rats by LC/LTQ-Orbitrap MS

**DOI:** 10.3390/molecules23051154

**Published:** 2018-05-11

**Authors:** Xinyu Zhang, Rui Zhao, Meng Chen, Tao Ma, Gaorong Wu, Nannan Xue, Guoliang Li, Hui Wang, Kang Fang, Wenxi Zhang, Penglong Wang, Haimin Lei

**Affiliations:** School of Chinese Pharmacy, Beijing University of Chinese Medicine, Beijing 100102, China; 20160931837@bucm.edu.cn (X.Z.); zr1012zr@126.com (R.Z.); chenmeng_0809@163.com (M.C.); mosesmatao@163.com (T.M.); gaorongwu09@163.com (G.W.); 18810612758@163.com (N.X.); liguoliangsx@163.com (G.L.); 15652387323@163.com (H.W.); 18364166994@163.com (K.F.); zhangwxnn@126.com (W.Z.)

**Keywords:** liquid chromatography-electrospray ionizationion trap mass spectrometry (LC/LTQ-Orbitrap MS), fragmentation regularities, ligustrazine derivative metabolites, neuroprotective activity

## Abstract

The neuroprotective evaluation of ligustrazine derivatives has become a research focus all over the world. A novel ligustrazine derivative, (3,5,6-Trimethylpyrazin-2-yl)methyl(E)-3-(4-((3,5,6-trimethylpyrazin-2-l)methoxy)phenyl)acrylate (T-CA), has shown protective effects against CoCl_2_-induced neurotoxicity in a differentiated PC12 cell model and middle cerebral artery occlusion (MCAO) model in our previous studies. However, nearly none of the parent drugs existed after rapid metabolism due to uncertain reasons. Thus, the fragmentation regularities of mass spectra, and metabolites, of T-CA in rats were examined using liquid chromatography-electrospray ionizationion trap mass spectrometry (LC/LTQ-Orbitrap MS) in this research. The main fragment ion, mass spectrum characteristics, and the structural information were elucidated. When compared with a blank sample, we identified five kinds of T-CA metabolites, including three phase I metabolites and two phase II metabolites. The results showed that the metabolic pathways of T-CA in rats via oral administration were hydrolysis (ether bond rupture, ester bond rupture), oxidation, reduction, glucose aldehyde acidification, etc. In addition, three main metabolites were synthesized and their structures were identified by superconducting high-resolution NMR and high-resolution mass spectroscopy (HR-MS). The neuroprotective activity of these metabolites was validated in a PC12 cell model. One of the metabolites (M2) showed significant activity (EC_50_ = 9.67 μM), which was comparable to the prototype drug T-CA (EC_50_ = 7.97 μM). The current study provides important information for ligustrazine derivatives, pertaining to the biological conversion process in vivo.

## 1. Introduction

It is important to evaluate the effects and toxicity of new drugs from metabolites, such as desloratadine, which showed a better anti-allergic activity than its parent drug loratadine [1]; amoxapine and its metabolites have the potential to alleviate irinotecan-induced diarrhea; and many microbial metabolites have the possibility to become therapeutic agents [2,3]. Tetramethylpyrazine is an alkaloid monomer extracted from the rhizome of *Ligusticum* chuanxiong, which can transport well across the blood–brain barrier, block the calcium channel, scavenge oxygen free radicals, affect the endothelium, and the synthesis process of nitric oxide [4,5,6,7,8]. In previous studies, tetramethylpyrazine was combined with small molecules of phenolic compounds, based on the traditional Chinese medicine combination principles [9,10,11,12,13,14,15]. According to different conjunctive positions and numbers of hydroxyls, over 100 novel ligustrazine-phenolic acid derivatives were synthesized and were proved to possess neuroprotective effects, both in vivo and in vitro [9,10,11,12,13]. The ligustrazine derivative (3,5,6-Trimethylpyrazin-2-yl)methyl(E)-3-(4-((3,5,6-trimethylpyrazin-2-l)methoxy)phenyl)acrylate (T-CA) was the best one among these tetramethylpyrazine derivatives because it showed an excellent neuroprotective activity. T-CA was evaluated in CoCl2-induced neurotoxic PC12 cell model, differentiated by NGF (nerve growth factor) in vitro, and MCAO (middle cerebral artery occlusion) rats model in vivo [11,14,16]. The internal absorption experiment indicated that the prototype compound was metabolized rapidly and few of it remained in plasma.

Numerous research on ligustrazine-phenolic acid derivatives, structural modification about their neuroprotective effect, inhibition of platelet aggregation, and vascular endothelial injury had been carried out [4,16,17]. Most of these conjugates were formed by ester bonds and amide bonds; however, there was little prior knowledge of stabilities and metabolism in vivo for such structures.

In this research, we intended to study the metabolic products of T-CA in vivo using high resolution mass spectrometry. The LC/LTQ-Oribitrap MS can obtain accurate mass without the standard substance by using high resolution in full-scan mode and a linear ion trap-electrostatic field, which is as high as 2 × 10^−6^ [18,19]. Its ability of rapid screening and structural confirmation of unknowns is a huge advantage to analyze compound composition in a complex system. Based on this, we identified five kinds of T-CA metabolites, including three phase I metabolites and two phase II metabolites. Three of these metabolites were synthesized and their structures were confirmed by HR-MS and NMR spectroscopy. Their neuroprotective activity was validated by cobalt chloride-induced neurotoxicity in differentiated PC12 cells. The results showed that metabolite M2 had significant activity, almost as same as the prototype drug (T-CA), which proved that drug action could be contributed from the metabolites. Thus, this analysis method provided an important direction for the evaluation of the ligustrazine derivative.

## 2. Results

### 2.1. The Mass Spectrometry and Lysis of T-CA

**Figure 1:**
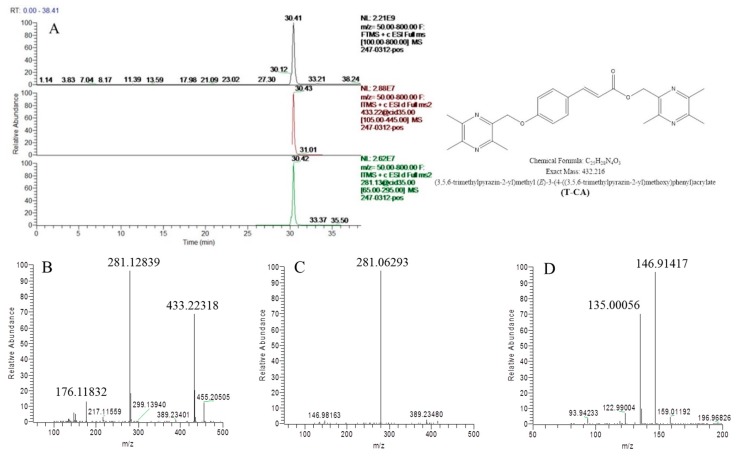
(**A**) Ion current chromatogram of (3,5,6-Trimethylpyrazin-2-yl)methyl(E)-3-(4-((3,5,6-trimethylpyrazin-2-l)methoxy)phenyl)acrylate (T-CA); (**B**) first-order full-scan mass spectrometry of T-CA; (**C**) secondary cleavage mass spectrometry of *m*/*z* 433.22318; (**D**) secondary cleavage mass spectrometry of *m*/*z* 281.06293.

The results in Figure 1 show that the T-CA excimer ion peak was *m*/*z* 433.22318 [M + H]^+^, and the fragments of the compound were *m*/*z* 281.12839 and 176.11832, respectively. The main characteristic of secondary mass spectrometry debris of *m*/*z* 433.22305 was *m*/*z* 281.06293, with the loss of a molecule (C_8_H_12_N_2_O). The fragment ion *m*/*z* 146.91417 and *m*/*z* 135.00056 were further crackate of fragment ion m/z 281.08398.

### 2.2. Identification of Plasma Metabolites of T-CA

**Figure 2:**
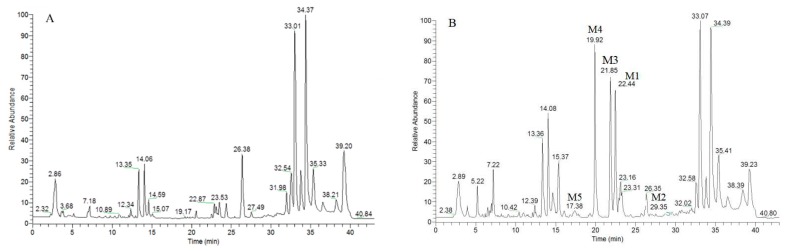
(**A**) The ion current chromatogram of control group; (**B**) the ion current chromatogram of administration group.

**Figure 3:**
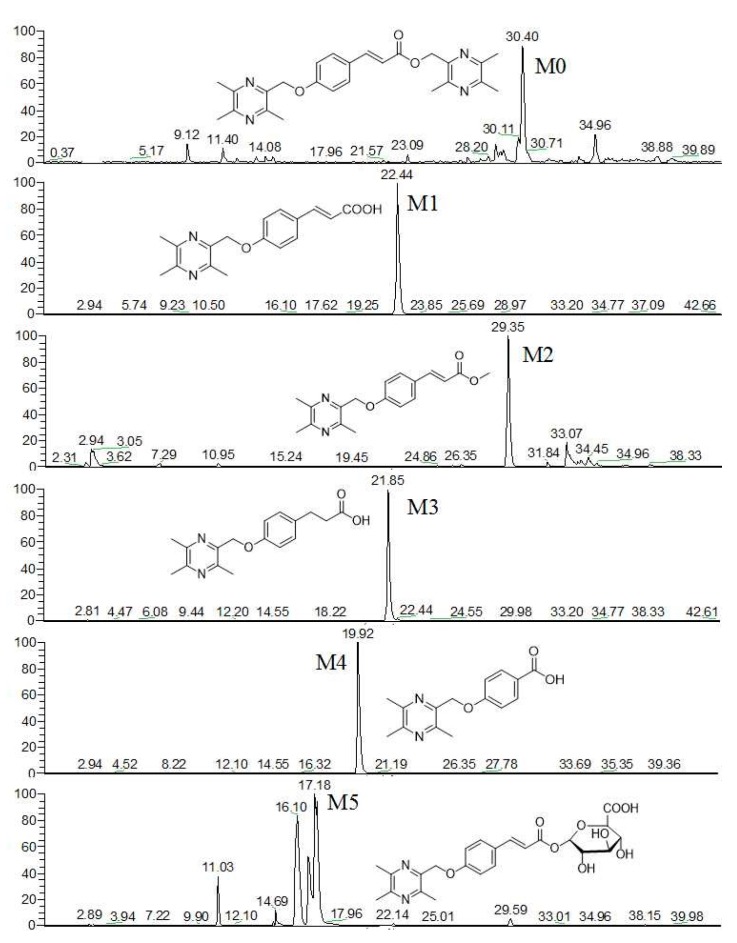
The ion flux and structure prediction of metabolites.

**Figure 4:**
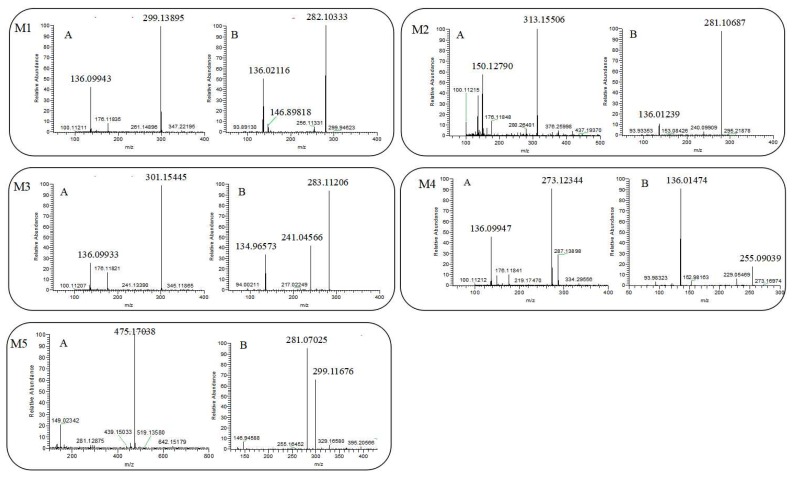
(**A**) Accurate mass spectrum of M1–M5; (**B**) the secondary cleavage of M1–M5.

Compared with blank plasma, there were three significant strong ion peaks (Figure 2) after T-CA was metabolized in vivo. The retention time peaks were 19.92, 21.85, and 22.44 min, respectively, which indicated that the compound T-CA could be rapidly absorbed through the intestinal tract into the blood. In addition, we achieved more metabolites through the extraction, which was verified via mass spectrometry (Figure 3 and Figure 4).

Five metabolites (M1–M5), as well as the prototype drug (M0), were detected in the plasma of the sample group residues. In the first-order full-scan mass spectrometry, the quasi-molecular ion of M0 was *m*/*z* 433.22418 [M + H]^+^, and the cleavage fragments were *m*/*z* 281.12912, 176.11862, 150.12801, and 136.09975. The chromatographic retention time was 30.40 min. As compared to the compound T-CA sample, the chromatographic retention time and the excimer ion were almost the same, therefore, M0 was determined as the parent drug.

#### 2.2.1. Identification of Plasma Metabolites M1

**Figure 5:**
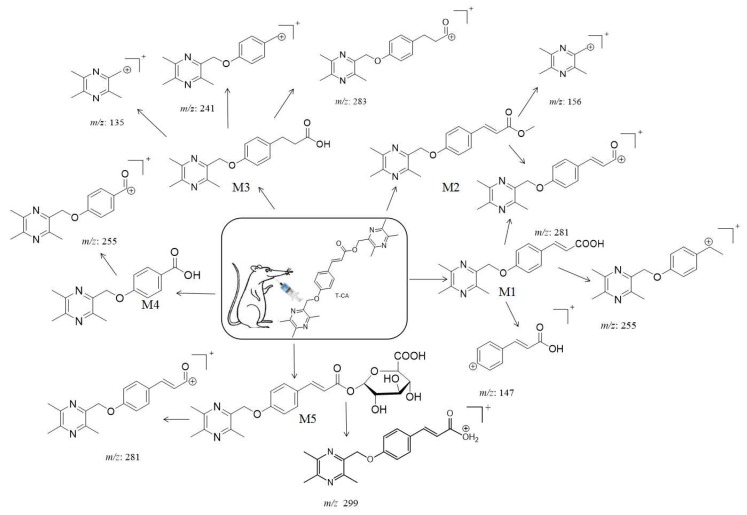
Proposed metabolic pathways of T-CA in rats.

M1 showed a [M + H]^+^ at *m*/*z* 299.13895 (C_17_H_19_N_2_O_3_), which was 136 Da less than T-CA, indicating that there was probably a loss of one molecule of ligustrazine. The chromatographic retention time of M1 was 22.44 min and its secondary debris ions were *m*/*z* 136.02116, 146.89818, and 282.10333. Based on the analysis of the information in Figure 4, we inferred that the structure for M1 was (E)-3-(4-((3,5,6-trimethylpyrazin-2-yl)methoxy)phenyl)acrylic acid and the possible cleavage method was showed in Figure 5 [20,21].

#### 2.2.2. Identification of Plasma Metabolites M2

M2 showed a [M + H]^+^ at *m*/*z* 313.15506 (C_18_H_20_N_2_O_3_), which is 14 Da more than M1 excimer ion (*m*/*z* 299.13895). We inferred that M2 was the methylation result of M1 carboxyl site and the structure is shown in Figure 4 [22,23]. The retention time of M2 was 29.35 min, and the main secondary mass fragments were *m*/*z* 136.01239 and *m*/*z* 281.10687. A possible method of cracking is shown in Figure 5. In addition, we synthesized the predicted structure M2 using the idea of drug design. Its structure was confirmed through data from mass spectrometry and the nuclear magnetic spectrum. Therefore, we concluded that M2 was methyl (E)-3-(4-((3,5,6-trimethylpyrazin-2-yl)methoxy)phenyl)acrylate. At the same time, the results provided the basis for the accuracy of M1 and M3′s structures.

#### 2.2.3. Identification of Plasma Metabolites M3

M3 showed a [M + H]^+^ at *m*/*z* 301.15445(C_17_H_20_N_2_O_3_), which was 2 Da more than the compound M1 excimer ion (*m*/*z* 299.13895), suggesting that M3 was probably the hydrogen reduction result of M1 in vivo (Figure 5). The chromatographic retention time of M3 was 21.85 min, which was one of the major metabolites produced in plasma. In addition, we could further confirm structure of M3 through its secondary debris ion: *m*/*z* 134.96573, 241.04566, 283.11206, the possible cleavage pathway is shown in Figure 4. Based on these data, the structure for M3 was identified as 3-(4-((3,5,6-trimethylpyrazin-2-yl)methoxy)phenyl)propanoic acid [24].

#### 2.2.4. Identification of Plasma Metabolites M4

M4 showed a [M + H]^+^ at *m*/*z* 273.12344 (C_17_H_20_N_2_O_3_), the retention time was 19.92 min, which was one of the definite metabolites. It was 26 Da less than M1 excimer ion (*m*/*z* 299.13895). Thus, we inferred that the double bond of M1 had broken before the occurrence of in vivo oxidation [25]. The possible structure of M4 was showed in Figure 4. In addition, we gave the cleavage pathway of M4 from its secondary cleavage fragment, which is shown in Figure 5. Therefore, 4-((3,5,6-trimethylpyrazin-2-yl) methoxy)benzoic acid was proposed as the structure for M4.

#### 2.2.5. Identification of Plasma Metabolites M5

M5 ([M + H^+^] *m*/*z* 475.17038, C_23_H_27_O_9_N_2_) was 176 Da (C_6_H_8_O_6_) heavier than M1, suggesting that M5 was a glucuronide conjugate. The diagnostic fragment ion at *m*/*z* 299.11676 (C_17_H_18_N_2_O_3_) was formed by the loss of glucuronide moiety from the protonated parent ion. As shown in Figure 4, other fragment ions at *m*/*z* 281.07025 and 146.94588 were consistent with the product ions of M1, indicating that the conjugation site was the C5 hydroxyl group [26,27]. M5 was confirmed as (2*R*,3*R*,4*R*,5*S*,6*S*)-3,4,5-trihyd-roxy-6-(((*E*)-3-(4-((3,5,6-trimethylpyrazin-2-yl)methoxy)phenyl)acryloyl)oxy)tetrahydro-2H-pyran-2-carboxylic acid And the retention time, accurate mass, elementary composition, and precision of metabolites are showed in Table 1.

**Scheme 1:**
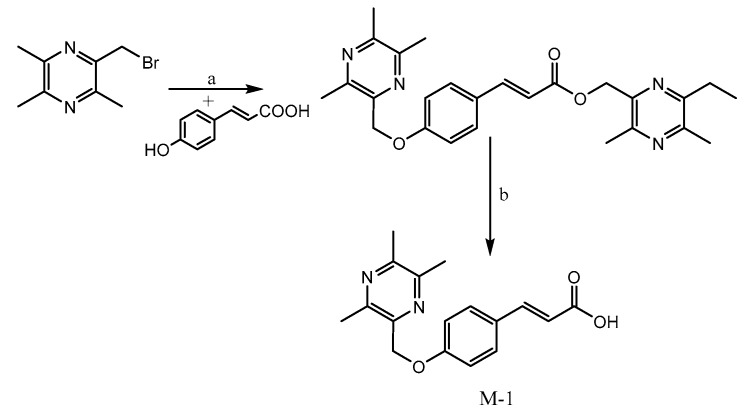
Reagents and conditions: (**a**) K_2_CO_3_, DMF, 85 °C, reflux, 2 h, (**b**) KOH, MeOH, 60 °C, reflux, 0.5 h.

**Scheme 2:**
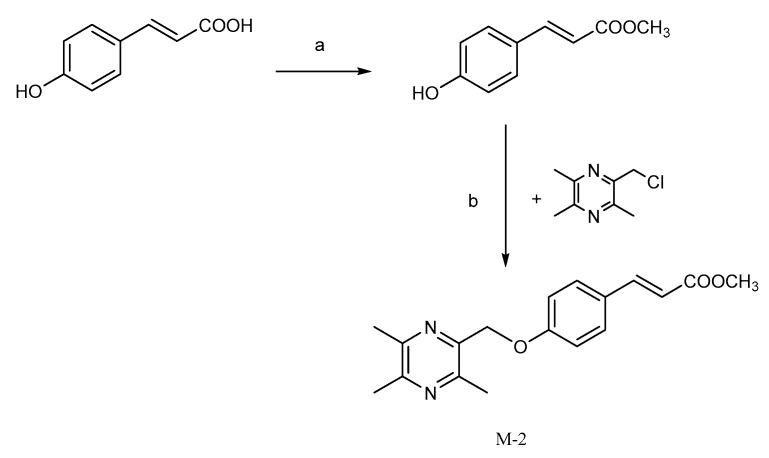
Reagents and conditions: (**a**) SOCl_2_, Methanol, r.t., 1 h. (**b**) K_2_CO_3_, DMF, 65 °C, reflux, 3 h.

**Scheme 3:**
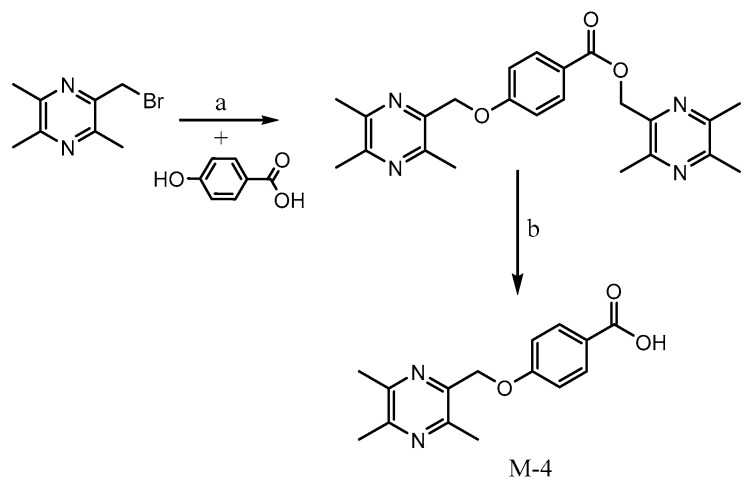
Reagents and conditions: (**a**) K_2_CO_3_, DMF, 85 °C, reflux, 2 h, (**b**) KOH, MeOH, 60 °C, reflux, 0.5 h.

According to these data, we inferred that five metabolites (M1–M5) were metabolized mainly by addition reaction, methyl esterification, and glucuronication. We synthesized three of them by chemical method and evaluated their biological activity. The results showed that all of them had neuroprotective activity especially metabolite M2; because its activity could reach the same level as T-CA, which provided an important direction for the discovery of novel compounds and the exploration of the T-CA onset pathways. Also, the amount of compound was recorded and collected for subsequent experimental exploration. The explicit structures and activity data are represented in the following schemes (Scheme 1, Scheme 2 and Scheme 3).

### 2.3. Neuroprotective Activity Test of Metabolites M1, M2, M4

**Figure 6:**
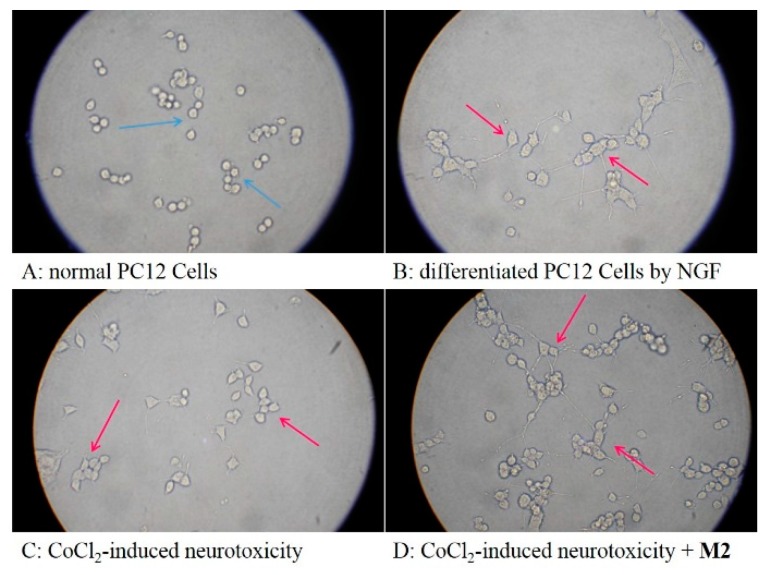
Effects of M2 on differentiated PC12 cell injury in morphology (×200); (**A**) undifferentiated PC12 cell; (**B**) PC12 cell differentiated by NGF; (**C**) CoCl_2_-induced differentiated PC12 cell; (**D**) CoCl_2_-induced neurotoxicity with M2 (60 μM).

As shown in Table 2, both T-CA and its metabolites had a neuroprotective effect under different concentrations, especially M2 presented a significant neuroprotective ability (EC_50_ = 9.67 μM), which was comparable to the prototype drug T-CA. Further, as shown in Figure 6, neuroprotective effects of M2 in PC12 cell model differentiated by NGF in morphology was observed through an optical microscope. Compared to PC12 cells injured by CoCl_2_ (Figure 6C), pretreatment of PC12 cells with M2 led to an alleviated morphological lesion (Figure 6D). Based on all the above results, we could conclude that the parent drug T-CA and its metabolites had neuroprotective activity both in vitro and in vivo.

## 3. Materials and Methods

### 3.1. Reagents, Chemicals and Animals

PC-12 cells (Peking Union Medical College Cell Resource Center, Beijing, China), RPMI 1640 medium, fetal bovine serum, horse serum, and nerve growth factor (Thermo Fisher company products, USA), Tetramethyl azo thiazole blue: MTT (China-Mainland, Sigma Aldrich, Beijing, China), Paraformaldehyde (Beijing Chemical Reagent Company, Beijing, China), and 5 mL of heparin lithium tube (GD050LH).

Compound T-CA, p-toluene sulfonyl chloride, ligustrazine, sulfoxide chloride, potassium carbonate, N, N-dimethyl formamide (DMF), tetrahydrofuran (THF), and anhydrous methanol (analytically pure or chemically pure), K_2_CO_3_, KOH, and Thionyl chloride (SOCl_2_).

Male SD rats (Beijing Viton Lihua Experimental Animal Technology Co., Ltd., Beijing, China)

### 3.2. Instrumentations

A series of main instrumentations were used in this study, such as tissue scissors, hemostatic clamps (Shanghai surgical instrument factory), an AEG-220 type electronic analytical balance (Shimadzu, Japan), vortex mixing apparatus Haimen Qilin Bell Instrument Manufacturing Co., Ltd. Model XW-80A), water bath nitrogen blowing instrument (Beijing Cheng Meng Albert Technology Co., Ltd., model CW-12) and KQ-500DE CNC ultrasonic cleaner (Kunshan City ultrasound Instrument Co., Ltd.). HPLC-HR-MS system was consists of several units: Thermo Accela Ultra-High Performance Liquid Chromatograph (Accela 600 Pump with Accela Open Autosampler and Quaternary Solvent Controller), Thermo LQT Orbitrap XL Mass Spectrometer, Xcalibur Workstation, ultra-pure helium as collision gas and high purity nitrogen for atomization. Structures were confirmed by AM-500 nuclear magnetic resonance instrument (Switzerland Bruker Company). In addition, Forma 3111 CO_2_ incubator, Multiskan GO full-wavelength microplate reader (Thermo Fisher, American), inverted microscope (OlympusIX71), and biological safety cabinet (HF) were used to finish the active test experiment.

### 3.3. Sample Preparation

Nine healthy male SD rats, weighing 280–300 g, were supplied by Beijing Vital River Laboratory Animal Technology Co., Ltd, Beijing, China. Rats were fasted for 12 h before receiving an oral administration of T-CA (dissolved in 0.5% CMC-Na) at a dose of 120 mg/kg body weight. The animals had access to water. The experiment was divided into two groups: Three males (control group) and six males (administration group: G1-G6). The control group was given intragastric administration of 0.5% CMC-Na. After 3 h, the rats were intraperitoneally injected with 10% chloral hydrate anesthesia (0.35 mL/100 g) before the blood sample was obtained from the heart region. Then the blood was injected into the heparin lithium centrifuge tube and centrifuged at 3000 rpm for 10 min. The supernatant was then transferred to 15 mL centrifuge tube, with three times the amount of acetonitrile, vortexed for complete mixing, and then was centrifuged at 10,000 rpm for 15 min. The supernatant was collected, with nitrogen blowing on a small amount of liquid. After being refolded with methanol, the samples were filtered with a 0.22 μm filter, and then stored at −20 °C for following analyses.

### 3.4. Liquid Chromatographic and Mass Spectrometer Conditions

A TC-C18 (4.6 mm × 250 mm, 5 μm, Agilent) column was used for analysis. The column temperature was kept at 30 °C. The mobile phases consisted of water containing 0.1% formic acid water (A) and acetonitrile (B). The gradient elution was done as follows: 90–40% A (0–28 min); 40–20% A (28–32 min); 20% A (32–37 min). The sample injection volume was 10 μL. The eluent flow rate was 1 mL/min.

For metabolite analysis, a full scan was run in the positive mode with a mass range from *m*/*z* 100 to 800 amu. The capillary pressure with the ESI ion source was +4 kV. The tapered voltage was +110 V and the ion source temperature and capillary temperature were, respectively, 300 °C and 350 °C. The collision gas was helium, the collision energy was 35 V and the sheath air flow and auxiliary air flow were 35 L/min and 10 L/min, respectively.

### 3.5. Experimental Procedure

#### 3.5.1. Preparation of M1 ((*E*)-3-(4-((3,5,6-trimethylpyrazin-2-yl)methoxy)phenyl)acrylic acid)

To verify the accuracy of the proposed structure, we synthesized metabolite M1 by esterification and hydrolysis. Compound 2-(bromome-thyl)-3, 5, 6-trimethylpyrazine (10.14 mmol), cumaric acid (5.07 mmol), and K_2_CO_3_ (5.0 mmol) were all added into DMF (30 mL), and then the mixture was stirred at 85 °C for 2 h. The crude product was extracted using ethylacetate and distilled under reduced pressure. The esterification product (1.85 mmol) and KOH (8 mL) were added to ethyl alcohol (20 mL), and then the mixture was stirred at 60 °C for 0.5 h. The pH value was adjusted to 3~4 and after being evaporated, the residue was eluted with silica gel, and the metabolite M1 was obtained. Compound M1 has been synthesized and reported by our laboratory [28]. M1: m.p.: 155.8–156.5 °C. 1H-NMR (500 MHz, CDCl3) δ: 7.52 (d, *J* = 16.5 Hz, 1H, -CH=), 7.28 (d, *J* = 8.5 Hz, 2H, Ar-H), 6.81 (d, *J* = 9.0 Hz, 2H, Ar-H), 6.08 (d, *J* = 16.0 Hz, 1H, =CH-), 5.33 (s, 2H, -CH_2_), 2.65 (s, 3H, -CH_3_), 2.58 (brs, 6H, -CH_3_). ^13^C-NMR (150 MHz, CDCl_3_) δ: 167.0, 159.1, 151.9, 149.5, 149.2, 145.5, 145.4, 145.0, 130.0, 126.2, 116.2, 113.9, 64.1 (-CH_2_), 21.5 (-CH_3_), 20.9 (-CH_3_), 20.4 (-CH_3_). HR-MS (ESI) *m*/*z*: 299.13885 [M + H]^+^, calcd. for C_17_H_18_N_2_O_3_ 298.13174.

#### 3.5.2. Preparation of M2 (methyl (*E*)-3-(4-((3,5,6-trimethylpyrazin-2-yl)methoxy)phenyl)acrylate))

To verify the accuracy of the proposed structure, we synthesized one of the metabolites, M2. Hydroxycinnamic acid (10.14 mmol) was added to anhydrous methanol (30 mL). After complete dissolution, thionyl chloride was slowly added dropwise under ice-cooling, while being stirred at 0 °C for 0.5 h, and then the temperature was slowly raised to room temperature. Then, the reaction solution was evaporated under reduced pressure. The reaction product was placed in a one-necked flask with potassium carbonate and N, N-dimethylformamide was added; the mixture was stirred at 65 °C for 3 h under nitrogen. Water was added to the reaction solution for dispersion before extraction with methylene chloride. After being evaporated, the residue was eluted with silica gel, and metabolite M2 was obtained. M2: m.p.: 124.0–124.4 °C. ^1^H-NMR (500 MHz, CDCl_3_) δ7.64~7.61 (d, *J* = 7.6Hz, 1H, Ar-H), 7.46~7.44 (d, *J* = 7.4 Hz, 1H, Ar-H), 7.01~6.99 (d, *J* = 7.3 Hz, 1H, Ar-H), 6.31~6.28 (d, *J* = 6.3 Hz, 1H, Ar-H), 5.16 (s, 2H, -CH_2_), 3.78, 2.63 (s, 3H, -CH_3_), 2.57 (s, 3H, -CH_3_), 2.51 (s, 3H, -CH_3_) ^13^C-NMR (150 MHz, CDCl_3_) δ 167.92, 160.58, 151.73, 150.20, 148.92, 145.45, 144.61, 129.93, 127.80, 115.78, 115.64, 115.43, 70.22 (-CH_2_), 51.81 (-OCH_3_), 21.94 (-CH_3_), 21.65 (-CH_3_), 20.85 (-CH_3_), HR-MS (ESI) *m*/*z*: 313.16403 [M + H]^+^, calcd. for C_18_H_20_N_2_O_3_.

#### 3.5.3. Preparation of M4 (4-((3,5,6-trimethylpyrazin-2-yl)methoxy)benzoic acid)

To verify the accuracy of the proposed structure, we synthesized one of the metabolites, M4. Compound 2-(bromome-thyl)-3,5,6-trimethy-lpyrazine (10.14 mmol), p-Hydroxybenzoic acid (5.07 mmol), and K_2_CO_3_ (5.0 mmol) were all added into DMF (30 mL), and then the mixture was stirred at 85 °C for 2 h. The crude product was extracted using ethylacetate and distilled under reduced pressure. The esterification product (1.97 mmol) and KOH (8 mL) were added into ethyl alcohol (20 mL), and then the mixture was stirred at 60 °C for 0.5 h. The pH value was adjusted to 3~4 and after being evaporated, the residue was eluted with silica gel, and the metabolite M4 was obtained. Compound M4 has been synthesized and reported by our laboratory [28]. M4: 160.7–161.4 °C. ^1^H-NMR (500 MHz, CDCl_3_) δ: 8.07 (d, *J* = 8.5 Hz, 2H, Ar-H), 7.07 (d, *J* = 8.5 Hz, 2H, Ar-H), 5.26 (s, 2H, -CH_2_), 2.63 (s, 3H, -CH_3_), 2.56 (s, 3H, -CH_3_), 2.56 (s, 3H, -CH_3_). ^13^C-NMR (150 MHz, CDCl_3_) δ: 171.2, 162.9, 151.6, 150.0, 148.9, 145.1, 132.3, 122.4, 114.6, 69.9 (-CH_2_), 21.7 (-CH_3_), 21.4 (-CH_3_), 20.5 (-CH_3_). HR-MS (ESI) *m*/*z*: 273.12234 [M + H]^+^, calcd. For C_15_H_17_N_2_O_3_ 273.11609.

### 3.6. Protective Effect on Injured PC12 Cells

PC12 cells were cultured in RPMI 1640 medium supplemented with horse serum (10% *v*/*v*), fetal bovine serum (5%, *v*/*v*), and penicillin-streptomycin (100 µ/mL) (Thermo Technologies, New York, NY, USA) at 37 °C when humidified with CO_2_ (5%). When the cells achieved the density of 80%, the original medium was removed and cells were cultured with the serum-free medium for 14 h. Then the cells were suspended in 1640 medium supplemented with 10% (*v*/*v*) fetal bovine serum, and seeded into poly-L-lysine-coated 96-well culture plates at 7 × 10^3^ cells/well, differentiated, and treated with 50 ng/mL nerve growth factor (NGF) for 48 h. Followed by these treatments, the differentiated PC12 cells were pretreated with various doses (60, 30, 15, 7.5, and 3.75 µg/mL) for 36 h. All measurements were performed after the cells were induced by CoCl_2_ (final concentration, 300 µM) for 12 h. The control-differentiated cells were not treated. CoCl_2_ was dissolved in RPMI 1640 medium. After MTT solution (20 µL, 5 mg/mL) was added to each well, the plate was incubated for a further 4 h at 37 °C. The optical density (OD) was measured at a wavelength of 490 nm using a BIORAD 550 spectrophotometer (Bio-Rad, Berkeley, CA, USA). The proliferation rates of damaged PC12 cells were calculated by the formula [OD490 (Sample)−OD490 (CoCl_2_)]/[OD490 (NGF) − OD490 (CoCl_2_)] × 100%.

## 4. Conclusions

In this study, the LC-MS^n^ detection method was established by analyzing T-CA and its metabolites in vivo. The results showed the main fragment ion peaks, mass spectral characteristics, and structural information of T-CA. There were five main metabolites of T-CA in rats: M1-M5. Three of these metabolites M1, M2, M4, were synthesized by a chemical method. The metabolites were validated according to the characteristics of the mass spectrometry. The PC12 cells model was used to verify that the metabolites had a certain neuroprotective activity; especially, metabolite M2 showed a strong activity (EC_50_ = 9.67 μM), which provided important information for further study of the T-CA biotransformation process and pre-modification. Studying the lead compounds as well as their metabolites at the same time is an important direction for drug discovery. Moreover, this research provided a reference for the study of ligustrazine derivative metabolism.

## Figures and Tables

**Table 1:** Retention time, accurate mass, elementary composition, and precision of metabolites.

Compound	t_R_/min	Species	Elemental Composition	*m*/*z*	RSD	Delta mmu
M0	30.40	[M + H]^+^	C_25_H_29_O_3_N_4_	433.22418	13.5	0.213
M1	22.44	[M + H]^+^	C_17_H_19_O_3_N_2_	299.13895	9.5	−0.199
M2	29.35	[M + H]^+^	C_18_H_21_O_3_N_2_	313.15506	9.5	0.481
M3	21.85	[M + H]^+^	C_17_H_21_O_3_N_2_	301.15445	8.5	−0.039
M4	19.92	[M + H]^+^	C_15_H_17_O_3_N_2_	273.12344	8.5	−0.109
M5	16.98	[M + H]^+^	C_23_H_27_O_9_N_2_	475.17038	11.5	0.063

**Table 2:** Neuroprotective activity of metabolites M1, M2, and M4.

Compound	Cell Proliferation Rate%					EC_50_
	60	30	15	7.5	3.75	
T-CA	102.94	89.83	71.09	27.89	39.02	7.97
M1	77.77	45.80	45.64	16.46	2.90	40.68
M2	89.95	82.06	75.97	42.04	20.69	9.67
M4	42.37	39.62	14.67	9.48	8.98	39.42
